# Downregulation of CDK5 signaling in the dorsal striatum alters striatal microcircuits implicating the association of pathologies with circadian behavior in mice

**DOI:** 10.1186/s13041-022-00939-2

**Published:** 2022-06-14

**Authors:** Hu Zhou, Jingxin Zhang, Huaxiang Shi, Pengfei Li, Xin Sui, Yongan Wang, Liyun Wang

**Affiliations:** State Key Laboratory of Toxicology and Medical Countermeasures, Beijing Institute of Pharmacology and Toxicology, Beijing, 100850 China

**Keywords:** Cyclin-dependent kinase 5 (CDK5), Dorsal striatum (DS), Neuronal projections, Motor activity, Circadian behavior

## Abstract

Dysfunction of striatal dopaminergic circuits has been implicated in motor impairment and Parkinson’s disease (PD)-related circadian perturbations that may represent an early prodromal marker of PD. Cyclin-dependent kinase 5 (CDK5) negatively regulates dopamine signaling in the striatum, suggesting a critical role of CDK5 in circadian and sleep disorders. Here, we used clustered regularly interspaced short palindromic repeats (CRISPR)/Cas9 gene editing to produce mice with a dorsal striatum (DS)-specific knockdown (KD) of the *Cdk5* gene (referred to as DS-CDK5-KD mice) and investigate its role in vivo. DS-CDK5-KD mice exhibited deficits in locomotor activity and disturbances in activity/rest behavior. Additionally, Golgi staining of neurons in the DS revealed that CDK5 deletion reduced dendrite length and the number of functional synapses, which was confirmed by significant downregulation of MAP2, PSD-95, and synapsin I. Correlated with this, DS-CDK5-KD mice displayed reduced phosphorylation of Tau at Thr181. Furthermore, whole-cell patch-clamp recordings of green fluorescent protein-tagged neurons in the striatum of DS-CDK5-KD mice revealed a decreased frequency of spontaneous inhibitory postsynaptic currents and altered excitatory/inhibitory synaptic balance. Notably, anterograde labeling showed that CDK5 KD in the DS disrupted long-range projections to the secondary motor cortex, dorsal and ventral thalamic nuclei, and basolateral amygdala, which are involved in the regulation of motor and circadian rhythms in the brain. These findings support a critical role of CDK5 in the DS in maintaining the striatal neural circuitry underlying motor functions and activity/rest associated with circadian rhythms that are perturbed in neurodegenerative disorders.

**Supplementary Information:**

The online version contains supplementary material available at 10.1186/s13041-022-00939-2.

## Introduction

Neurodegenerative disorders, such as Parkinson’s disease (PD) and Alzheimer’s disease (AD), are increasing in prevalence with the aging of the population. In addition to the classical motor symptoms of neurodegenerative disorders in older adults, high rates of sleep disorders, such as insomnia, hypersomnia, sleep apnea are likely caused by damage to sleep-controlling regions of the brain. PD is a devastating neurodegenerative disorder characterized pathologically by the loss of dopaminergic neurons in the substantia nigra (SN) pars compacta (SNpc) [1]. The dorsal striatum (DS) is the main recipient of dopaminergic innervation from the SNpc, and accordingly, the impact of its loss on striatal microcircuitry has been extensively studied in PD [2, 3]. Dysfunction of striatal dopaminergic neural circuits has been implicated in the pathophysiology of both motor and non-motor symptoms in PD [4, 5], and sleep disorders are more common in PD than in AD. It is estimated that rest/activity cycle disturbances in PD emerge when approximately 60% of nigral neurons are lost, and dopaminergic striatal content is reduced by 80% [6, 7]. Thus, it is important to recognize and properly manage these sleep disorders because treatment may improve the symptoms of a neurodegenerative condition and substantially improve quality of life [8, 9].

Traditionally, dopamine (DA) has been associated with wake-promoting activity. Amphetamines promote wakefulness by enhancing DA release and preventing its reuptake by DA transporters [10]. Modafinil, a wakefulness inducer, has also been linked to dopaminergic activity [11, 12]. The striatum is the main recipient of dopaminergic innervation from the SNpc and is composed primarily of GABAergic medium spiny projection neurons (MSNs) that express the DA receptors D_1_ and D_2_ [13, 14]. Dopamine and cAMP-regulated phosphoprotein 32 (DARPP-32) is a highly enriched cytosolic protein in MSNs and is considered an important integrator of striatal cellular excitability and synaptic transmission [15]. DA exerts bidirectional control on the phosphorylation state of DARPP-32 at Thr34; D_1_ receptors stimulate, and D_2_ receptors inhibit this phosphorylation. Additionally, cyclin-dependent kinase 5 (CDK5) phosphorylates DARPP-32 at Thr75, leading to the dephosphorylation of DARPP-32 at Thr34 [16, 17]. Therefore, CDK5 negatively regulates DA signaling in the striatum, indicating a functional role of CDK5 in regulating the rest/activity cycle. In addition, CDK5 modulates dendritic spine formation and cortical neurotransmission. CDK5 dysfunction is associated with several neurodegenerative disorders, such as AD and PD [18, 19]. For example, CDK5 phosphorylates the NMDA receptor subunit NR2B to modulate synaptic transmission [20] and phosphorylates postsynaptic density protein 95 (PSD-95) to promote synaptic PSD-95 clustering and glutamate transmission [21]. These findings indicate that aberrant hypoactivation of CDK5 may contribute to neural circuitry disorders in human and rodent brains. In mouse models of PD, 1-methyl-4-phenyl-1,2,3,6-tetrahydropyridine (MPTP) treatment results in higher levels of CDK5 and its specific activator, the P25 complex, in dopaminergic neurons, leading to neuronal death [22]. Furthermore, MPTP is toxic to dopaminergic neurons in the SNpc, causing irreversible changes to striatal DA levels and PD symptoms, including a perturbed daily activity/rest behavior [23–25]. Thus, we predict that CDK5 in DS involves in the maintenance of wakefulness and the control of movement, although the mechanisms underlying these effects are not completely understood.

Here, we aimed to assess the role of CDK5 in the regulation of circadian rest/activity behavior using clustered regularly interspaced short palindromic repeats (CRISPR)/Cas9 gene editing to generate dorsal striatum (DS)-specific CDK5 knockdown (KD) mice (referred to as DS-CDK5-KD mice). The DS was selected because it is a major area of DA involvement in the early stages of motor processing, and DS disturbance may produce the well-documented behavioral changes associated with cerebral neurodegenerative disorders [26]. In addition to assessing motor activity, we examined the changes of wake/sleep behaviors in DS-CDK5-KD mice. Electrophysiological and anterograde labeling studies were performed to examine whether CDK5 KD impairs cellular excitability and synaptic transmission in the striatal network, which appears to play a key role in the pathophysiology of sleep dysfunction in mice. This article is intended to clarify the possible mechanism of CDK5 in wake/sleep behavior associated with circadian rhythm.

## Materials and methods

### Animals

Eight-week-old male C57BL/6 mice, initially weighing 18–20 g (Vital River Laboratories, Beijing, China), were group-housed in a controlled environment at 18–22 °C and 40–60% humidity, with a 12/12 h light/dark cycle and access to food and water ad libitum. Enrichment was provided with nestlet shredding. Cages (32 × 22 × 17 cm, eight mice per cage) were changed every week by designated facility staff. All mice were group-housed for 1 week prior to use in experiments and handled daily to minimize the effects of handling stress. All experiments were performed in accordance with the National Institutes of Health *Guide for the Care and Use of Laboratory Animals* (NIH Publications No. 80-23) and approved by the Animal Care and Use Committees of the Beijing Institute of Pharmacology and Toxicology. Efforts were made to minimize the number of animals used for each experiment.

### In vivo KD of the *Cdk5* gene in the mouse DS

**Fig. 1:**
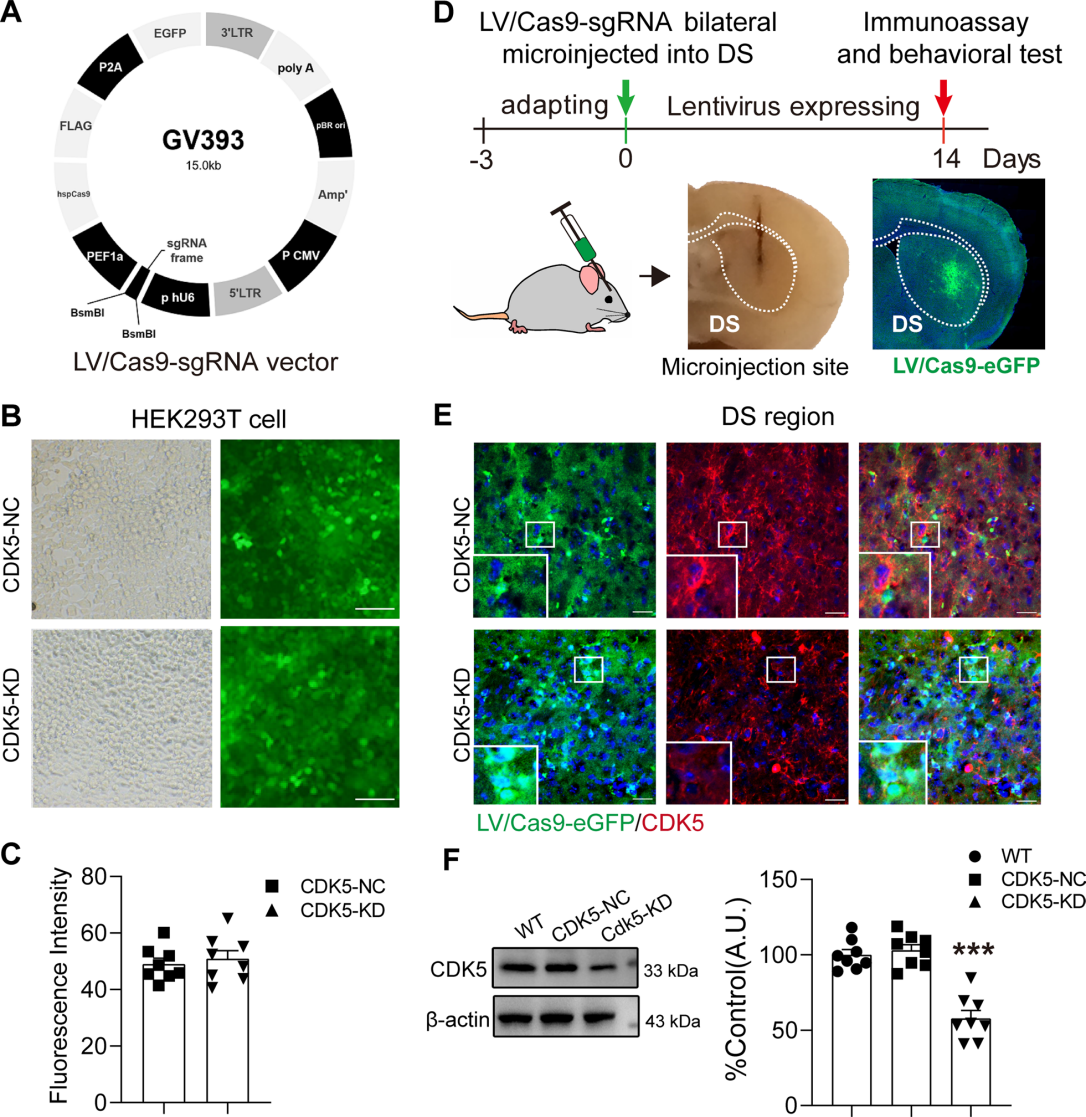
Lentivirus expressing *Cdk5*-sgRNA reduces CDK5 protein levels in the DS. Immunoassays were performed 14 days after LV/Cas9-sgRNA microinjection. **A** Schematic of the LV/Cas9-sgRNA expression vector. **B** HEK293 cells transduced with 10 μL LV/Cas9-*Cdk5*-sgRNA (2 × 10^8^ pfu/mL) and eGFP expression of HEK293-T evaluated using a fluorescence microscope. Scale bars = 200 μm. **C** The transfection rate of the virus, indicating a well-established effect relationship between the transfect rate and LV/Cas9-Cdk5-sgRNA virus. **D** Experimental timeline and schematic of microinjection sites in the mouse brain. LV/Cas9-sgRNA were highly expressed in the DS region, as indicated by GFP (green) under fluorescence microscopy. **E** Representative immunofluorescence image of CDK5 staining (red) in the dorsal striatum (DS) of CDK5-NC and CDK5-KD mice. The enlarged image shows multiple mCherry-labeled neurons in the DS (scale bar = 10 μm). **F** Representative western blot assay of CDK5 expression in the DS. Band intensity of CDK5 relative to β-actin is shown as the mean ± SEM, *n* = 8 independent experiments. One-way ANOVA, Tukey’s multiple comparisons test, *p < 0.05, **p < 0.01, ***p < 0.001 vs WT

To efficiently KD mouse CDK5 in the DS, *Cdk5*-targeting single-guide RNAs (sgRNAs) were designed (Fig. 1A). Lentiviral (LV) vectors (p24 stock solutions ranging between 200 and 300 ng/µL and approximately 2 × 10^8^ transducing units/mL) co-expressing a P2A promoter-driven enhanced green fluorescent protein (eGFP) and a U6 promoter-driven small single-guide RNA (sgRNA), either specifically directed against *Cdk5* mRNA (LV-*Cdk5*-sgRNA) or a negative control (LV-NC-sgRNA), were produced by GeneChem Co., Ltd (Shanghai, China). The sequence used for targeted editing of *Cdk5* was 5ʹ-TCAGCTTCTTGTCACTATGC-3ʹ, and the non-specific NC sequence was 5ʹ-CGCTTCCGCGGCCCGTTCAA-3ʹ. Standard procedures and LV/Cas9-sgRNA validation in vivo were reported previously [21]. Briefly, HEK293T cells were plated in 6-well plates (5 × 10^5^ cells/well) and cultured in Dulbecco’s Modified Eagle Medium with 10% fetal bovine serum at 37 °C in 5% CO_2_. Once cells were completely attached, they were transduced with 10 μL LV-*Cdk5*-sgRNA or LV-NC-sgRNA (2 × 10^8^ pfu/mL). The medium was replaced after 48 h, and the cells were further incubated until 48 h as required. For in vitro validation, C57BL/6J mice were anesthetized with pentobarbital sodium [50 mg/kg, intraperitoneal (i.p.), Sigma-Aldrich, St. Louis, MO, USA] and mounted on a stereotaxic apparatus (Zhongshidichuang Science and Technology Development Co., Ltd, Beijing, China). Mice were randomly divided into wild-type (WT), LV-NC-sgRNA (CDK5-NC), and LV-*Cdk5*-sgRNA (CDK5-KD) groups, and the respective LV vectors were bilaterally microinjected into the DS. For microinjection, we used the following two bilateral sites in the DS (1.5 µL per site, 3 µL total) with stereotaxic coordinates (in mm) according to the modified stereotaxic mouse atlas of Paxinos and Franklin [27]: (1) anterior/posterior (A/P) + 1.1, medial/lateral (M/L) ± 1.2, dorsal/ventral (D/V) − 3.2 and (2) A/P + 0.9, M/L ± 2.0, D/V − 3.2. The delivery rate was 0.33 µL/min using a UMP3 microsyringe injector and Micro4 controller (World Precision Instruments, Sarasota, FL, USA). The needles were left in place for 3 min to allow for full delivery of the solutions and then slowly retracted. Next, the scalp was sutured with 7-0 Vicryl sutures (ETHICON Co., Ltd, New Jersey, USA).

### Experimental design and behavioral tests

Mice were screened 3 days before use, and mice with abnormal behavior were excluded. Mice were randomly divided into WT, DS-CDK5-NC, and DS-CDK5-KD groups, with 30 mice in each group. Fourteen days after LV transduction, 20 mice from each group were selected for behavioral testing. After the behavioral tests were completed, mice were deeply anesthetized with ether and sacrificed by decapitation for immunoassays (immunofluorescent and western blot). The remaining mice were used for Golgi staining, electrophysiology assays, and anterograde labeling. All behavioral experiments were stared at 9:00 am. Each behavioral test was performed on the same animals with an interval of 24 h between tests. The testing apparatus was cleaned with a hypochlorous acid solution between subjects. The experimenters were blinded to grouping and drug treatment.

#### Locomotor activity test

A locomotor activity test was used to assess spontaneous locomotor activity and arousal in mice. The method used was similar to a previously published protocol [28]. Briefly, animals were placed individually in a square arena (50 × 50 × 80 cm) with a Plexiglas floor and walls (Zhongshidichuang Science and Technology Development Co., Ltd) and allowed to move freely. After a 5-min habituation period, all animal locomotor activities were recorded with AnyMaze software (Stoelting Inc., Wood Dale, USA), and the distance traveled during a 60-min period was recorded and analyzed.

#### Wheel-running behavioral test

Mice were housed in temperature-controlled circadian cabinets (Zhongshidichuang Science and Technology Development Co., Ltd) within polypropylene cages (33.2 × 15 × 13 cm) containing a metal running wheel (35-cm diameter). The experiment started at 6:00 am and ended at 6:00 pm on the next day (total 48 h; LD condition: 12 h-light/12 h dark cycle × 2; DD condition: 48 h dark). Mice were acclimatized to the running wheel for 1 h prior to assessment. Rest/activity behavior were monitored with a ClockLab data collection system (Version 3.603, Actimetrics, Wilmette, IL, USA) by determining the number of electrical closures triggered by wheel rotations. The number and duration of wheel running by mice were the main indicators. The α/ρ ratio is the ratio of wheel running duration (α) to wheel stopping duration (ρ). Cage changes were performed in 48-h intervals. Wheel-running activity was monitored over 48 h and analyzed using ClockLab Analysis software (Actimetrics Software).

### Golgi staining and dendritic spine measurements

Golgi-Cox staining was performed using a Rapid Golgi Stain Kit (FD Neuro Technologies, Ellicott City, MD, USA) following the manufacturer’s instructions. Briefly, brains were quickly removed, rinsed, and incubated in a mix of solution A/B for 14 days in the dark at room temperature. Then, solution A/B was changed to solution C for 3 days. Coronal sections of the DS (200 μm thick) were cut (ranging from 0.7–1.2 mm anterior to bregma; two sections per animal) on a freezing microtome (Leica, Wetzlar, Germany) and mounted onto gelatinized slides. After sections were dried in the dark, they were reacted in solutions D and E for 10 min and dehydrated sequentially in 50%, 75%, 95%, and 100% ethanol. Finally, sections were cleared in xylene and cover slipped with resinous mounting medium.

For dendritic spine measurements, the DS region was identified at low power (100× magnification), and MSNs were traced at 250× (final magnification) using the camera lucida technique on an Olympus light microscope (Model BX51) equipped with a drawing attachment and the 8-bit ImageJ plugin Neuron J. Dendrite length and branching were measured by Sholl’s analysis of ring intersections. A series of concentric rings at 20 µm increments printed on a transparent grid was centered over the soma. The total number of intersections between each ring and dendritic branches was counted and converted to estimates of dendrite length as a function of distance from the soma (i.e., for each 20-µm segment) and overall dendrite length. Spine density was measured manually in the stacks using the ImageJ Plugin Cell Counter. Three to five dendrite segments per slice, with each dendrite segment ranging from 20 to 30 μm in length, were used for spine density analysis. Spines were marked in the appropriate focal plane, preventing any double counting of spines.

### Immunohistochemistry

After the last behavioral test, mice were euthanized, and striatum sections were prepared for immunohistochemistry. Briefly, striatal sections (30 μm) were cut with a vibratome (VT1000 S, Leica) and collected in phosphate-buffered saline (PBS) as free-floating sections. Sections were rinsed three times in PBS and permeabilized and blocked in PBS containing 0.3% Triton X-100 and 5% normal goat serum (Pierce Biotechnology, Rockford, IL, USA) for 1 h at room temperature. Sections were then washed in PBS and incubated overnight at 4 °C with an anti-CDK5 antibody (1:400; Cat# ab40773, Abcam, Cambridge, England) and anti-GAD antibody (1:400; Cat# ab26116, Abcam), which was detected with an anti-rabbit Alexa Fluor 594 secondary antibody (1:200; Absorption Peak: 590 nm, Emission Peak: 617 nm; Cat# ZF-0516, ZSGB Biotech, Jiangsu, China) and anti-mouse Alexa Fluor 488 secondary antibody (1:200; Absorption Peak: 493 nm, Emission Peak: 519 nm; Cat# ZF-0512, ZSGB Biotech). To identify nuclei, sections were counterstained in mounting medium containing 4ʹ,6-diamidino-2-phenylindole (DAPI) (Sigma-Aldrich). Finally, sections were mounted and examined using a fluorescence microscope (Zeiss, Oberkochen, Germany or Leica, Germany).

### Western blotting

Western blot assays were used to measure protein levels. After the last behavioral test, mice were euthanized with pentobarbital sodium salt (50 mg/kg, i.p.). The striatum was dissected and homogenized in radioimmunoprecipitation assay lysis buffer [10 mM PBS, pH 7.4, containing 1% NP-40, 0.5% sodium deoxycholate, 0.1% SDS, and complete protease inhibitor cocktail (Roche, Basel, Switzerland)]. Tissue dissection was performed in the same time window for all groups. For western blotting, solubilized proteins (50 µg per sample) were resolved on SDS-PAGE gels and transferred onto polyvinylidene fluoride membranes (Millipore, Bedford, MA, USA). The membranes were blocked with 5% non-fat milk diluted in TBST at room temperature for 1 h and then incubated overnight at 4 °C with the following primary antibodies: anti-CDK5 mouse monoclonal antibody (1:1000; Cat# ab40773, Abcam), anti-MAP2 rabbit polyclonal antibody (1:1000; Cat# ab183830, Abcam), anti-synapsin 1 mouse monoclonal antibody (1:1000; Cat# MAN894, Millipore), anti-PSD-95 rabbit polyclonal antibody (1:500 dilution, Cat# ab12093, Abcam), anti-phospho-Tau (Ser202) rabbit polyclonal antibody (1:800; Cat# 11834s, Cell Signaling Technology, MA, USA), anti-phospho-Tau (Thr181) rabbit polyclonal antibody (1:800; Cat# 12885s, Cell Signaling Technology), anti-Tau mouse monoclonal antibody (1:1000; Cat# MAB3420, Millipore), and anti-β-actin mouse monoclonal antibody (1:1000; AF0003, Beyotime). The membranes were then washed with TBST and incubated with the appropriate peroxidase-conjugated secondary goat anti-rabbit/mouse IgG (1:2000; Cat# AB-2301/ZB-2305, ZSGB-Bio, Beijing, China) for 1 h at room temperature. An ECL detection kit (Thermo Fisher Scientific, Waltham, MA, USA) was used, and immunoreactive bands were quantified on an imaging system (ProteinSimple, San Jose, CA, USA). β-actin was used as the loading control.

### Anterograde labeling

To characterize the dopaminergic projections to different brain regions, adeno-associated virus (AAV)-hSyn-mCherry-IRES-WGA-Cre was packaged (SunBio Biomedical Technology Co. Ltd., Shanghai). On day 14 after *Cdk5*-sgRNA injection, the AAV-hSyn-mCherry-IRES-WGA-Cre was bilaterally microinjected into the center of the original virus injection points (coordinates: A/P + 1.0 mm, M/L + 1.8 mm, D/V − 3.2 mm). Animals were anesthetized throughout surgery with pentobarbital sodium (50 mg/kg, i.p.). Following craniotomy, a microsyringe was lowered into the brain using the above coordinates, and 500 nL of AAV-hSyn-mCherry-IRES-WGA-Cre were injected over a 10-min period [29]. Thirty days later, mice were deeply anesthetized and immediately perfused transcardially with normal saline followed by 4% paraformaldehyde. Brains were removed, post-fixed overnight in the same solution, cryoprotected by immersion in 30% sucrose, and then frozen in dry ice-cooled methyl butane. Serial coronal cryostat sections (40 μm) through the whole brain were cut with a vibratome (VT1000 S, Leica), rinsed in PBS, counterstained with the nuclear dye DAPI (0.2 mL; Zsbio, Beijing, China), and mounted on slides. Images were captured with a microscope slide scanner (Pannoramic SCAN II, 3DHistech, Ltd., Budapest, Hungary). Coronal sections of the cortex, striatum, thalamus, and basolateral amygdala (BLA) were confirmed in the microscope slide scanner. CaseViewer 2.3 software (3DHistech, Ltd.) was used for image observation and analysis.

### Electrophysiology

Mice were anesthetized with pentobarbital sodium (50 mg/kg, i.p.), and brains were removed. Striatal slices (300 μm) were cut in ice-cold cutting solution [(in mM) 124 NaCl, 2.8 KCl, 1.25 NaH_2_PO_4_, 2 CaCl_2_, 1.25 MgSO_4_, 26 NaHCO_3_, 10 glucoses; pH 7.3; bubbled with 95% O_2_/5% CO_2_] using a vibrating tissue slicer (MA752, Campden Instruments, Loughborough, UK). Coronal slices were submerged for 30 min at 32 °C in artificial cerebrospinal fluid (ACSF) [(in mM) 126 NaCl, 2.5 KCl, 26.2 NaHCO_3_, 1.25 NaH_2_PO_4_, 2 CaCl_2_, 1.5 MgSO_4_, 10 d-glucose, 5 sodium ascorbate; pH 7.3; bubbled with 95% O_2_/5% CO_2_] at 295–300 mOsm/L. All reagents were from Sigma-Aldrich (St. Louis, MO, USA) unless otherwise indicated. Following this recovery period, slices were transferred to room-temperature ACSF until recording.

Patch pipettes were prepared from borosilicate glass (Sutter Instrument Company, Novato, CA, USA) using a P-97 Flaming/Brown micropipette puller (Sutter Instrument Company) and had a resistance of 6–8 MΩ when filled with the following intracellular solution [(in mM) 130 CsCl, 10 NaCl, 0.25 CaCl_2_, 2 MgCl_2_, 5 EGTA, 10 HEPES, 10 glucose, 2 Mg-ATP, 0.3 Na_2_-GTP]. The pH of the pipette solution was adjusted to 7.3 with 1 mM CsOH, and osmolarity was adjusted to 285–290 mOsm/L. A low-power objective (4×) was used to identify the DS region, and a 40× water immersion objective (NIR Apo, Nikon, Japan) coupled to an infrared differential interference contrast microscope with a fluorescence system and a CCD camera was used to visually identify, patch, monitor, and record CDK5-KD neurons in the DS. MSNs were identified according to previously determined membrane characteristics and firing properties [30]. Recording in normal current-clamp or voltage-clamp modes was performed with a Digidata 1440A digitizer, an Axon 200B amplifier, and Clampex 10.2 software (all from Molecular Devices, San Jose, CA, USA) at room temperature. Fast and slow capacitance compensation were performed after tight-seal (> 1 GΩ) formation. During whole-cell recordings, series resistance was monitored and compensated (80–90%) periodically. When the series resistance of a neuron was above 50 GΩ or changed by more than 25%, it was excluded from further analysis. Data were filtered at 2 kHz and acquired at a sampling rate of 10 kHz. Access resistance and leak currents were monitored, and recordings were rejected if these parameters changed significantly during data acquisition.

For miniature excitatory postsynaptic current (mEPSC) recordings, the oxygenated ACSF contained the GABA receptor antagonist (+)-bicuculline (10 μM; Sigma-Aldrich) and the voltage-gated sodium channel blocker tetrodotoxin (TTX) (1 μM; Abcam) to abolish inhibitory postsynaptic current (IPSC) events and action potentials. For spontaneous IPSC (sIPSC) recordings, the oxygenated ACSF contained the competitive NMDA receptor antagonists 6-cyano-7-nitroquinoxaline-2, 3-dione (CNQX) (20 μM; Sigma-Aldrich) and l-(+)-2-amino-5-phosphonopentanoic acid (l-AP5) (50 μM; Tocris, Ellisville, MO, USA), as well as TTX (1 μM) to abolish excitatory postsynaptic current events and action potentials. The sections were superfused with the oxygenated ACSF solutions at a rate of 1.2 mL/min. Spontaneous activity was recorded 5 min after the whole-cell mode was obtained for at least 3 min. Data were analyzed using Clampfit 10.2 (Molecular Devices), OriginPro 2018 (Origin Lab, Washington, MA, USA), and/or GraphPad Prism 7 (GraphPad, San Diego, CA, USA).

### Statistical analysis

GraphPad Prism 8.0 software (GraphPad Software, Inc, La Jolla, CA, USA) was used for behavior analyses, western blotting, fluorescence imaging, and Golgi staining. Clampex 10.2 software (Molecular Devices, Union City, CA, USA) was used for electrophysiology analysis, and the Kolmogorov–Smirnov test was used to compare the cumulative distributions of frequency and amplitude between groups. Differences between groups were determined by one-way analysis of variance followed by Tukey’s multiple comparisons test. All results are expressed as the mean ± standard error of the mean. The number of samples/subjects per experiment is noted in the corresponding figure legend. A *p*-value < 0.05 was considered statistically significant.

## Results

### Motor impairment after LV delivery of *Cdk5*-sgRNA into the DS of mice

We generated DS-CDK5-KD mice using the CRISPR–Cas9 system, and the efficiency of LV/Cas9-*Cdk5*-sgRNA transduction was examined in vitro and ex vivo. For the in vitro study, HEK293 cells were transduced with 10 μL LV/Cas9-Cdk5-sgRNA and LV/Cas9-NC-sgRNA (2 × 10^8^ pfu/mL) for 96 h. We found that transduction with LV/Cas9-sgRNA resulted in high eGFP expression (Fig. 1B), and there was no significant difference in the viability of HEK293-T cells transduced with LV/Cas9-*Cdk5*-sgRNA and LV/Cas9-NC-sgRNA (Fig. 1C). In vitro validation demonstrated that LV/Cas9-*Cdk5*-sgRNA could be used in further experiments. For the in vivo study (Fig. 1D), LV/Cas9-*Cdk5*-sgRNA was highly expressed at the microinjection sites of DS regions, as indicated by GFP (green) under fluorescence microscopy. Meanwhile, immunofluorescence studies revealed abundant CDK5 staining (red) in virus-transfected cells (green) in the DS transfected with LV/Cas9-NC-sgRNA, whereas CDK5 staining (red) was greatly reduced in DS-CDK5-KD mice throughout the 14 days after LV/Cas9-sgRNA injection (Fig. 1E), indicating the effectiveness of this LV. Accordingly, we extracted protein from eGFP-positive areas in the WT, DS-CDK5-NC, DS-CDK5-KD mice for western blot analysis. CDK5 expression in the DS of mice injected with LV/Cas9-NC-sgRNA did not change significantly, whereas CDK5 expression was significantly decreased in mice injected with LV/Cas9-*Cdk5*-sgRNA [Fig. 1F; WT, 100.0 ± 3.51%, LV/Cas9-NC-sgRNA, 103.10 ± 3.77%, LV/Cas9-*Cdk5*-sgRNA, 57.89 ± 5.27%, F (2, 21) = 35.25, *p* < 0.0001]. Taken together, the immunostaining and western blot analyses showed that LV/Cas9-sgRNA efficiently knocked down CDK5 in DS neurons by day 14 after injection.

**Fig. 2:**
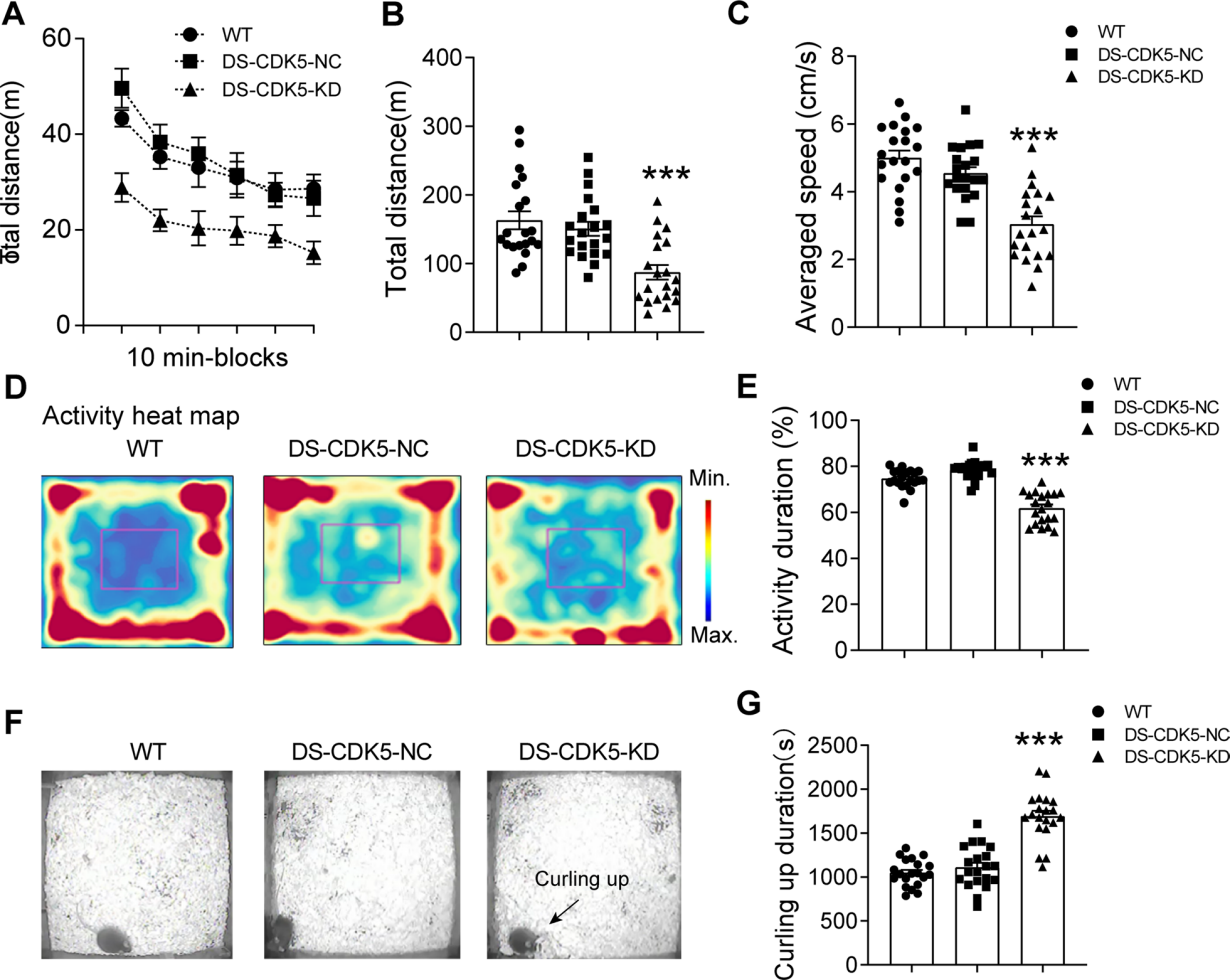
DS-specific CDK5-KD affects the motor activity of mice. **A** The locomotor activity test was performed 14 days after CDK5 or NC-sgRNA microinjection. Movement was recorded for 60 min and reported as distance in centimeters (cm) per 10 min. **B**, **C** ANOVA analysis of total distance traveled (**B**) and averaged speed (**C**) in the locomotor test of WT, DS-CDK5-NC, and DS-CDK5-KD mice. **D** Activity heatmap of mice in the locomotor test. Blue indicates low activity, and red indicates high activity. **E** Statistical analysis of activity duration (%) in the locomotor test chamber. **F** WT and DS-CDK5-NC mice exhibited normal activity with free-walking, as in the left and middle panels, whereas DS-CDK5-KD mice spent more time immobile and curled up, as in the right panel, during the test period. **G** Quantitative analysis of the curling up behavior in the locomotor test chamber. Data are represented as the mean ± SEM, *n* = 20 mice per group. One-way ANOVA, Tukey’s multiple comparisons test, *p < 0.05, **p < 0.01, ***p < 0.001 vs WT

We then examined whether reduced CDK5 expression in the DS altered behavioral performance. Locomotor activity was monitored at day 14 after LV/Cas9-sgRNA injection. The distance plot of DS-CDK5-KD mice in 10-min intervals revealed severe spontaneous general activity abnormalities (Fig. 2A). Statistical analysis showed that both the total distance traveled [Fig. 2B; WT, 163.1 ± 13.11.62 m vs DS-CDK5-KD, 87.37 ± 10.60 m; F (2, 57) = 12.74, *p* < 0.001] and the total velocity [Fig. 2C; WT, 5.00 ± 0.21 cm/s vs DS-CDK5-KD, 3.04 ± 0.27 cm/s; F (2, 57) = 23.67, *p* < 0.001] were significantly reduced in DS-CDK5-KD mice. In addition, the activity heatmap revealed that DS-CDK5-KD mice preferred to travel in the peripheral area more than the central area of the arena (Fig. 2D). Consistent with this, statistical analysis showed that the activity duration (% total 60-min) was significantly decreased in DS-CDK5-KD mice [Fig. 2E; WT, 74.57 ± 0.87% vs DS-CDK5-KD, 64.86 ± 1.87%, F (2, 57) = 59.37, *p* < 0.001]. In addition, DS-CDK5-KD mice spent more time nest building and curled up (Fig. 2F) during the test period [Fig. 2G; WT, 1048 ± 34.43 s vs DS-CDK5-KD, 1693 ± 62.73 s, F (2, 57) = 48.11, *p* < 0.001], which may simply be a matter of comfort as animals seek warmth to prepare for sleeping. In contrast, both WT and DS-CDK5-NC mice exhibited normal free-walking activity. In line with the immunostaining and western blot analyses showing efficient KD of CDK5 in the DS, the altered behavior indicated that CDK5 deficiency in the DS affects the DS neural network that controls motor behavior.

### Circadian change of wheel-running activity is altered in DS-CDK5-KD mice

**Fig. 3:**
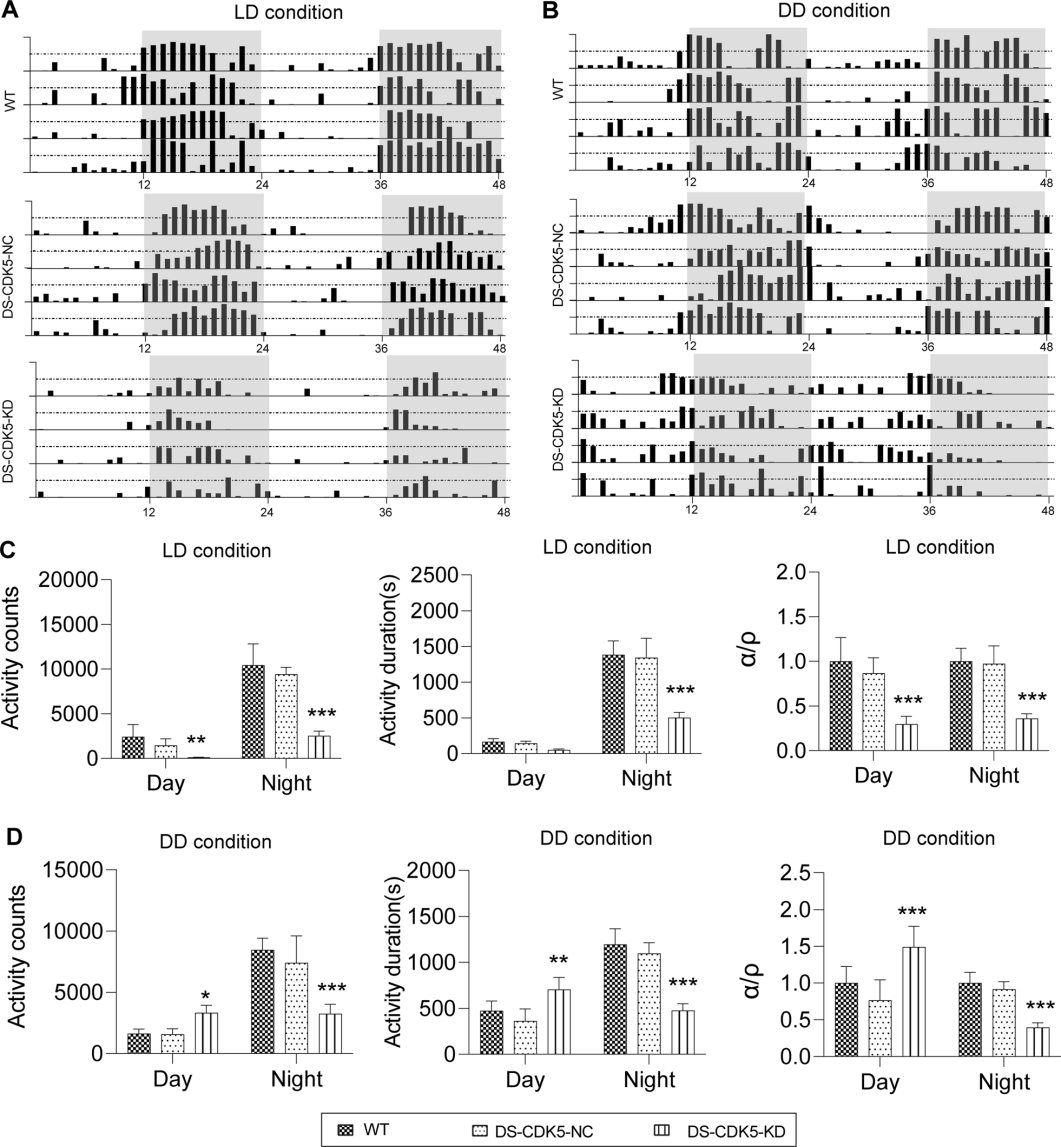
Altered circadian wheel-running activity correlates with the loss of CDK5 in the DS in mice. **A** Representative actograms showing wheel-running activity during entrainment in a 12/12 h light/dark (LD) cycle, in which gray shading represents the dark periods. **B** Representative actograms showing wheel-running activity under a 12:12 dark/dark (DD) cycle, in which gray shading represents the periods of the subjective night. Note one line represent running duration from an exercised mouse in 48 h, and data were obtained from four animals per group. **C** Activity counts, activity durations, and α/ρ ratio in a 12:12 night/day cycle under LD conditions for WT, DS-CDK5-NC, and DS-CDK5-KD mice. **D** Activity counts, activity durations, and α/ρ ratio in a 12:12 night/day cycle under DD conditions for WT, DS-CDK5-NC, and DS-CDK5-KD mice. Data are represented as the mean ± SEM, *n* = 12 mice per group. One-way ANOVA, Tukey’s multiple comparisons test, *p < 0.05, **p < 0.01, ***p < 0.001 vs WT

To examine the DS-specific CDK5-driven 48 h activity/rest behavior associated with circadian change, wheel-running activity was continuously monitored for 48 h under LD and DD conditions. Hourly activity profiles are shown in Fig. 3A (LD conditions) and B (DD conditions). WT and DS-CDK5-NC mice sustained normal rhythms of activity/rest behavior with free movement during the night but exhibited a lower overall activity during the daytime in both LD and DD conditions. Under normal LD conditions (Fig. 3C), DS-CDK5-KD mice exhibited lower activity counts in running wheel tests during the day and night (day: WT, 2398.13 ± 1383.83 vs DS-CDK5-KD, 100.53 ± 28.80, *p* < 0.01; night: WT, 10,427.91 ± 2393.71 vs DS-CDK5-KD, 2533.07 ± 532.26, *p* < 0.001) and shorter activity duration during the day and night (day: WT, 166.19 ± 44.57 s vs DS-CDK5-KD, 49.56 ± 14.27 s, *p* < 0.001; night: WT, 1381.32 ± 197.83 s vs DS-CDK5-KD, 503.13 ± 74.22 s, *p* < 0.001). It is well known that the α/ρ ratio [the ratio of running duration (α) to rest duration (ρ)] is positively correlated with the circadian period [31, 32]. Accordingly, the α/ρ ratio of DS-CDK5-KD mice was significantly decreased under the LD condition (day: WT, 1.00 ± 0.27 vs DS-CDK5-KD, 0.29 ± 0.08, *p* < 0.001; night: WT, 1.00 ± 0.14 vs DS-CDK5-KD, 0.36 ± 0.05, *p* < 0.001).

Interestingly, after closer examination of the daily patterns of activity under abnormal DD conditions (Fig. 3D), we observed that the activity counts were significantly increased during periods of the subjective day (activity counts: WT, 2255.78 ± 1196.37 vs DS-CDK5-KD, 3515.18 ± 1482.33, *p* < 0.05) but dramatically reduced during periods of the subjective night (activity counts: WT, 8461.97 ± 951.62 vs DS-CDK5-KD, 3244.14 ± 776.65, *p* < 0.001) in DS-CDK5-KD mice. Notably, compared with littermate WT mice, a remarkable difference in circadian activity duration was observed in DS-CDK5-KD mice under DD conditions (subjective day: WT, 473.77 ± 106.95 s vs DS-CDK5-KD, 704.26 ± 131.69 s, *p* < 0.01; subjective night: WT, 1194.21 ± 170.17 s vs DS-CDK5-KD, 476.23 ± 75.19 s, *p* < 0.001). The α/ρ ratio during periods of the subjective day was significantly higher (day: WT, 1.00 ± 0.23 vs DS-CDK5-KD, 1.49 ± 0.40, *p* < 0.001), and the α/ρ ratio during periods of the subjective night was significantly lower (night: WT, 1.00 ± 0.14 vs DS-CDK5-KD, 0.40 ± 0.07, *p* < 0.001). This reversal in the α/ρ ratio (a higher α/ρ during the subjective day and a lower α/ρ during the subjective night) suggested disruption of activity/rest behavior associated with circadian system in DS-CDK5-KD mice.

### CDK5 deficiency causes morphological alterations in MSN dendrites and spines in the DS

**Fig. 4:**
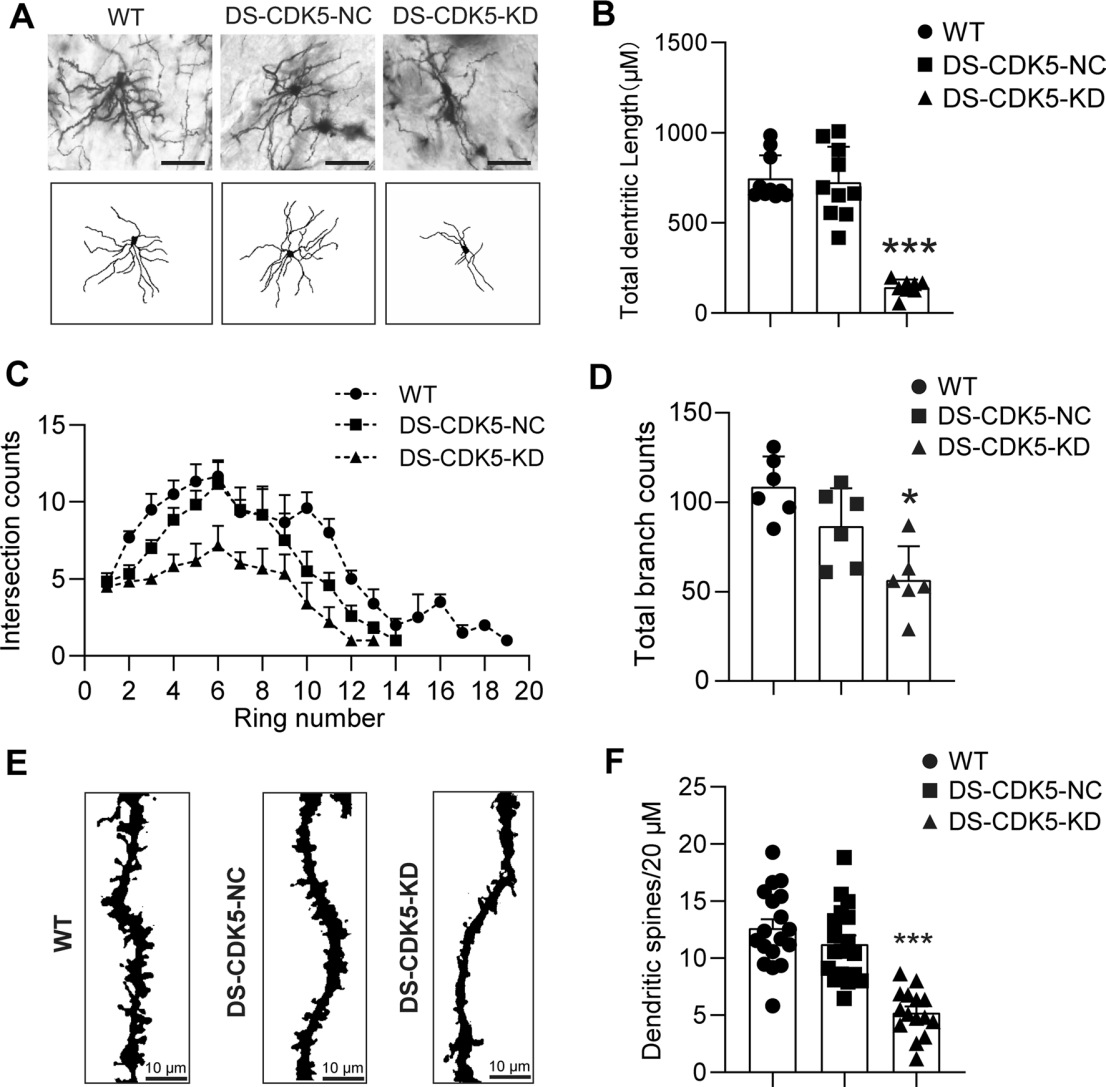
Effects of CDK5 knockdown on dendrite length and spine density of DS neurons. **A** Upper panel: Representative Golgi staining of dendrites in the DS of WT, DS-CDK5-NC, and DS-CDK5-KD mice; Lower panel: 8-bit neuron tracing images. Scale bar = 20 µm. **B**–**D** Quantification of dendrite length and branching of MSN tracing images. **E** Representative photographs of MSN dendrite segments from the DS of WT, DS-CDK5-NC, and DS-CDK5-KD mice. **F** Cell counter for spine density analysis. *n* = 10–20 MSNs/segment from four slices of two animals per group. ANOVA analysis showing a reduction in dendrite length, branching, and spine density in DS-CDK5-KD mice compared with those in WT and DS-CDK5-NC mice. Data are represented as the mean ± SEM. One-way ANOVA, Tukey’s multiple comparisons test, *p < 0.05, **p < 0.01, ***p < 0.001 vs WT

CDK5 is required for radial neuronal dendrite and spine maintenance [33], and CDK5 dysregulation may dramatically affect striatal-dependent brain function. Here, we performed an additional experiment to rule out the detrimental effects of CDK5 KD on the MSNs in the striatum. As indicated by HE, TUNEL, and Nissl staining (Additional file 1 and 2), when compared with those in controls, the results showed that there were not significantly increased neuronal apoptosis and loss in *Cdk5*-deficient MSNs studied in ex vivo. Furthermore, we showed the stratification of apical MSN morphology in the striatum of WT and DS-CDK5-NC mice, and significant changes in neuronal morphology were observed in DS-CDK5-KD mice, as determined by Golgi staining (Fig. 4A). Dendrite lengths in WT (746.3 ± 40.81 μm, *n* = 20 MSNs) and DS-CDK5-NC (725.0 ± 62.49 μm, *n* = 12 MSNs) mice were not significantly different. However, in DS-CDK5-KD mice, dendrite length (142.8 ± 15.00 μm, *n* = 12 MSNs) was significantly reduced [Fig. 4B; F (2, 25) = 48.91, *p* < 0.0001]. By concentric circle (Sholl’s) analysis, we found a significant decrease in dendritic branching in DS-CDK5-KD mice (Fig. 4C). Impaired dendritic branching occurred proximal (rings 3–8) and then distal to the soma (rings 9–20), indicating significantly decreased MSN neurite arborization in DS-CDK5-KD mice. Moreover, total dendritic branching was significantly reduced in DS-CDK5-KD mice (56.50 ± 7.68, *n* = 12 MSNs) compared with that in WT (108.5 ± 6.98, *n* = 20 MSNs) and DS-CDK5-NC (86.50 ± 8.66, *n* = 20 MSNs) mice [Fig. 4D; F (2, 15) = 11.18, *p* = 0.0011]. CDK5 KD in the DS also impaired MSN spine generation and significantly decreased dendritic spine density. Representative sections of dendritic spines are shown for each group in Fig. 4E. The dendritic spine density per 20 μm was significantly reduced in DS-CDK5-KD mice (5.23 ± 0.52, *n* = 15 segments) compared with that in WT (12.61 ± 0.79, *n* = 16 segments) and DS-CDK5-NC (11.21 ± 0.77, *n* = 16 segments) mice [Fig. 4F; F (2, 48) = 27.37, *p* < 0.0001].

**Fig. 5:**
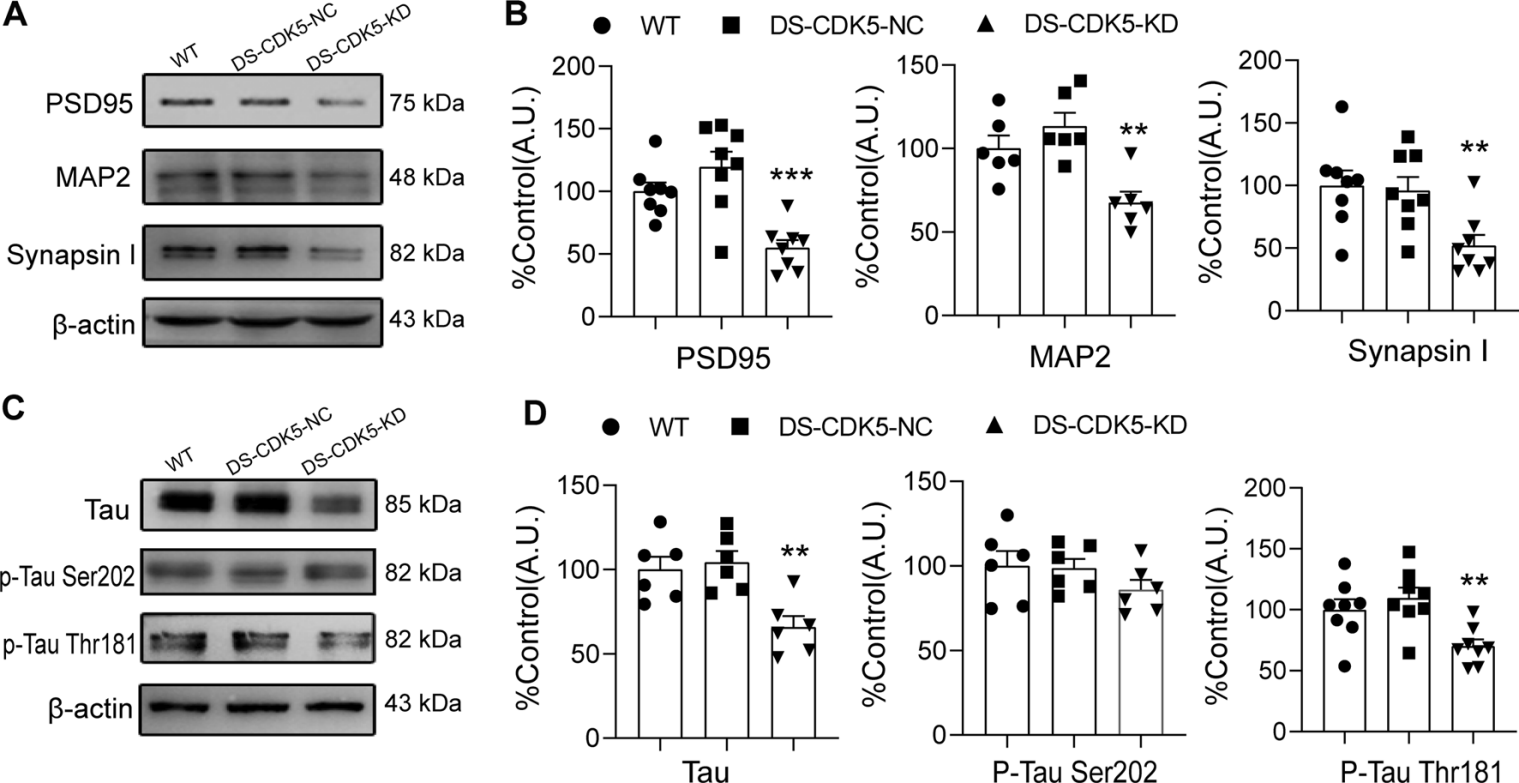
CDK5 deficiency reduces the expression of PSD-95, MAP2, synapsin 1 and Tau phosphorylation in the DS. **A** Representative blots of PSD-95, MAP2 and synapsin I from the DS of WT, DS-CDK5-NC, and DS-CDK5-KD mice. **B** ANOVA analysis of relative protein levels of PSD-95, MAP2 and synapsin 1. **C** Representative blots of total Tau and phosphorylated Tau at Thr181 and Ser202 from the DS of WT, DS-CDK5-NC, and DS-CDK5-KD mice. **D** ANOVA of relative Tau, p-Tau Ser202, and p-Tau Thr181 levels. Data are represented as the mean ± SEM, *n* = 6–8 independent experiments. One-way ANOVA, Tukey’s multiple comparisons test. *p < 0.05, **p < 0.01, ***p < 0.001 vs WT

Because the surgery and LV did not damage the DS (Additional file 2), we performed western blotting to determine the levels of synaptic and spine formation proteins (Fig. 5A), including MAP2 (Microtubule associated protein 2), synapsin 1 (presynaptic proteins), and PSD-95 (postsynaptic protein-95). Statistical analysis revealed that DS-CDK5-KD mice had significantly decreased levels of PSD-95 [Fig. 5B; WT, 100.0 ± 7.04% vs DS-CDK5-KD, 54.83 ± 16.47%; F (2, 21) = 13.82, *p* = 0.0001], MAP2 [Fig. 5B; WT, 100.0 ± 7.65% vs DS-CDK5-KD, 67.56 ± 6.53%; F (2, 15) = 10.15, *p* = 0.0016], and synapsin 1 [Fig. 5B; WT, 100.04 ± 12.02% vs DS-CDK5-KD, 52.09 ± 8.40%; F (2, 21) = 6.350, *p* = 0.007]. Next, we examined Tau protein and its phosphorylation sites Thr181 and Ser202 (Fig. 5C), which are closely related to synapse reconstruction and neuronal morphology maintenance. Compared with those in WT mice, total Tau protein levels were significantly reduced in DS-CDK5-KD mice [Fig. 5D; WT, 100.0 ± 7.56% vs DS-CDK5-KD, 65.82 ± 6.58%; F (2, 15) = 9.150, *p* = 0.005]. Interestingly, there were no changes in the phosphorylation of Tau at Ser202 in DS-CDK5-KD mice [Fig. 5D; WT, 100.0 ± 8.76% vs DS-CDK5-KD, 85.96 ± 5.82%; F (2, 15) = 1.280, *p* = 0.31]. However, we found a significant decrease in the phosphorylation of Tau at Thr181 in DS-CDK5-KD mice compared with that in WT mice, indicating a reduction in the phosphorylation activity of CDK5 at Thr181 [Fig. 5D; WT, 100.0 ± 8.69% vs DS-CDK5-KD, 70.19 ± 5.47%; F (2, 21) = 7.055, *p* = 0.0045]. These data indicate that CDK5 downregulation in the DS perturbs dendrite branching and spine formation and the phosphorylation of Tau at Thr181, and CDK5 plays a key role in synaptic transmission. Together, these results demonstrate that CDK5 KD disrupts morphology and induces biochemical changes in DS neurons.

### CDK5 deficiency affects inhibitory synaptic transmission in the DS

**Fig. 6:**
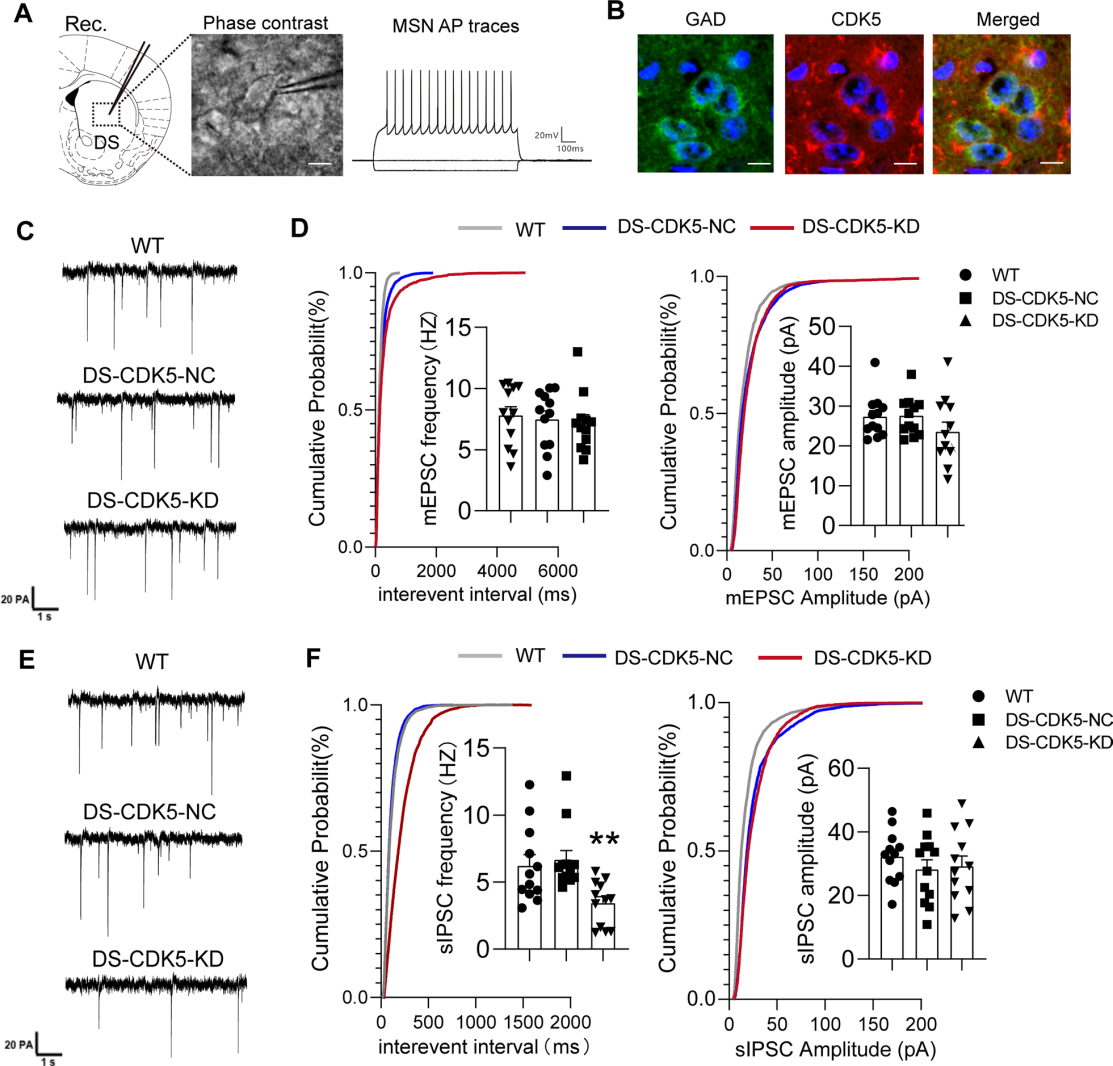
CDK5 knockdown perturbs synaptic transmission in the DS. **A** Schematic depiction of electrode placements and action potential properties recorded from MSNs in the DS. **B** Immunohistochemical identification of recorded neurons is GAD-expressing MSNs in DS slices. Scale bar: 10 μm. **C** Representative mEPSC traces recorded from MSNs from WT, DS-CDK5-NC, and DS-CDK5-KD mice. Scale bars = 20 pA/1 s. **D** Cumulative histograms and quantification of mEPSC frequency and amplitude from (**C**). **E** Representative sIPSC traces recorded from MSNs of WT, DS-CDK5-NC, and DS-CDK5-KD mice. Scale bars = 20 pA/1 s. **F** Cumulative histograms and quantification of mEPSC frequency and amplitude from (**E**). Data are represented as the mean ± SEM, *n* = 12 MSNs from four mice per group. One-way ANOVA, Tukey’s multiple comparisons test. *p < 0.05, **p < 0.01, ***p < 0.001 vs WT

Deletion of CDK5 dramatically affects the morphology of MSNs in the DS, which may subsequently affect the neuronal signal transduction that underlies behavior. Therefore, we recorded mEPSCs and sIPSCs using whole-cell techniques. Moreover, action potential properties were used to recognize MSNs in the DS region, as indicated in Fig. 6A. Electrodes were placed in MSNs which were confirmed by double-labeling for CDK5 with glutamate decarboxylase (GAD) after recording, as indicated in Fig. 6B. The mEPSCs reflect the presynaptic release of neurotransmitters from vesicles [34]. Representative mEPSC traces were showed at Fig. 6C. In DS-CDK5-KD mice, there was no change in the frequency of mEPSCs [Fig. 6D; WT, 7.48 ± 0.69 Hz vs DS-CDK5-KD, 7.80 ± 0.70 Hz, F (2, 33) = 0.20, *p* = 0.82] or the average amplitude [Fig. 6D; WT, 27.32 ± 1.54 pA vs DS-CDK5-KD, 26.53 ± 2.40 pA, F (2, 33) = 1.44, *p* = 0.25]. mEPSCs recorded in the presence of TTX showed no changes in amplitude and frequency. Therefore, we recorded sIPSCs without TTX and studied the impact of CDK5 KD on inhibitory synapses and inhibitory changes in the loop (Fig. 6E). sIPSC amplitude was unaffected in DS-CDK5-KD mice [Fig. 6F; WT, 32.17 ± 2.39 pA vs DS-CDK5-KD, 29.17 ± 3.26 pA, F (2, 33) = 0.51, *p* = 0.61]. However, the frequency of sIPSCs was reduced in DS-CDK5-KD mice [Fig. 6F; WT, 6.22 ± 0.82 Hz vs DS-CDK5-KD, 3.41 ± 0.48 Hz; F (2, 33) = 6.59, *p* = 0.0039]. Given that the frequency of sIPSCs reflects the release of presynaptic GABA [35], CDK5 likely enhances GABA receptor-mediated neurotransmission. These findings indicate that dysregulation of CDK5 in the striatum may alter inhibitory synaptic transmission within the DS in a manner that is consistent with the morphological and behavioral deficits.

### CDK5 deficiency in the DS reduces long-range projections to the secondary motor cortex (M2), thalamic nucleus, and BLA nucleus

**Fig. 7:**
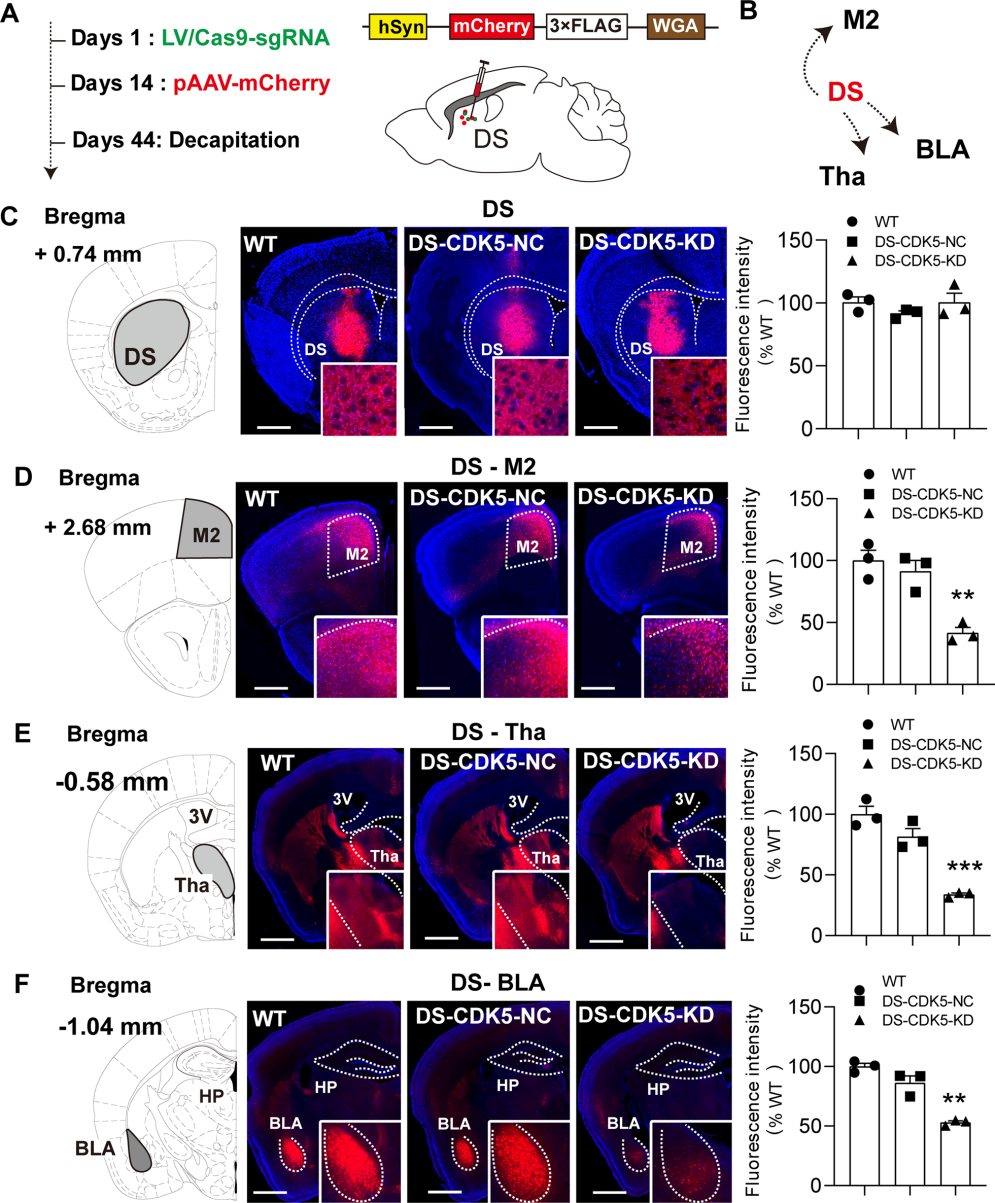
CDK5 deficiency reduces long-range projections from the DS to the M2, thalamus, and BLA. **A** Experimental procedure. On the 14th day after LV/Cas9-sgRNA injection, AAV-hSyn-mCherry (red) was unilaterally injected into the DS. **B** Scheme of the simplified DS projection pathways. The DS pathway has been simplified, and only the representative DS projection targets are displayed. M2, secondary motor cortex; Tha: thalamic nucleus; BLA, basolateral amygdaloid nucleus. **C** Coronal section and analysis graph showing anterograde labeling 30 days after AAV injection in the DS (scale bar = 100 μm); the enlarged image shows multiple mCherry-labeled neurons in the DS (scale bar = 10 μm). **D–F** M2 (**D**), thalamus (**E**), and BLA (**F**) areas are labeled with mCherry projected from the DS. Data are represented as the mean ± SEM, *n* = 3 sections from three mice per group. One-way ANOVA, Tukey’s multiple comparisons test. *p < 0.05, **p < 0.01, ***p < 0.001 vs WT

To examine whether CDK5 KD in the DS affects striatal-dependent brain function, we performed anterograde tracing with AAV-hSyn-mCherry to label fiber tracts that project from the DS (Fig. 7A). Figure 7B illustrates a scheme of the simplified DS projection pathways. This sensitive neuroanatomical tract tracing technique can be used to visualize neuronal projections, including dendritic arbors [36]. All injections were centered in the DS [coordinates: A/P + 1.0 mm, M/L + 1.8 mm, D/V − 3.2 mm from bregma] and visualized using fluorescence microscopy to evaluate injection accuracy. Striatal sections obtained after anterograde AAV injection showed that most striatal neurons expressed the mCherry tracer in the DS region (Fig. 7C). Four weeks after AAV-hSyn-mCherry microinjection, whole brain sections were used to trace long-range axonal connections from the DS to the M2, dorsal thalamic nuclei, and BLA, which are regions closely associated with motor function and circadian rhythm regulation. Figure 7D–F illustrates the pattern of anterograde labeling observed in the M2, thalamus, and BLA in WT, DS-CDK5-NC, and DS-CDK5-KD mice. In WT and DS-CDK5-NC mice, most anterogradely labeled cells were located in the M2, thalamic nuclei, and BLA, which revealed the close connection of all three brain regions with the DS. In contrast, the viral tracers were unevenly and weakly present in the M2, thalamus, and BLA of DS-CDK5-KD mice. Bar graphs of mCherry fluorescence in the M2, thalamus, and BLA show that fluorescence intensities in all three brain areas were significantly reduced in DS-CDK5-KD mice compared with those in WT mice [mCherry fluorescence in the M2 (Fig. 7D): WT, 100.0% ± 6.49% vs DS-CDK5-KD, 33.91% ± 1.21%; F (2, 6) = 40.68, *p* = 0.003; mCherry fluorescence in the thalamus (Fig. 7E): WT, 100.0% ± 2.78% vs DS-CDK5-KD, 52.97% ± 1.40%; F (2, 9) = 40.81, *p* = 0.0003; mCherry fluorescence in the BLA (Fig. 7F): WT, 100.1% ± 8.27% vs DS-CDK5-KD, 41.72% ± 4.37%; F (2, 6) = 18.43, *p* = 0.0027]. These findings indicate that CDK5 is important for neural connectivity between the DS and the M2, thalamus, and BLA. The DS is the main integration station of the basal ganglia, and CDK5 may have a critical role in maintaining the neural circuits associated with motor function and circadian rhythms in the DS.

## Discussion

The regulation of DA/DARPP-32 neurotransmission by CDK5 in the striatum has been previously evaluated, and the aberrant hypoactivation of CDK5 may contribute to the neural circuitry underlying circadian disorders in the human and rodent brain. However, the role of CDK5 in circadian regulation is unknown. Therefore, we used an LV-based CRISPR/Cas9 system to efficiently knock down the *Cdk5* gene in the DS of mice. DS-CDK5-KD mice exhibited behavioral deficits in locomotor activity and disturbed daily rest/activity cycles, along with dendrite and spine morphological abnormities and impaired basal GABA-mediated sIPSCs in the DS. Furthermore, CDK5 deficiency reduced long-range connections from the DS to the M2, thalamus, and BLA. These findings provide insight into the involvement of striatal CDK5 in circadian modulations.

The study of sleep and alertness in neurodegenerative disorders is extremely challenging. In our study, using CRISPR/Cas9-mediated gene editing, we efficiently achieved selective knockout of the *Cdk5* gene in the DS of mice (Fig. 1E and F). Fourteen days after viral vector injection, WT and DS-CDK5-NC mice displayed normal locomotor behaviors during the rest/activity cycle. In contrast, DS-CDK5-KD mice exhibited marked reductions in total distance traveled and average moving speed. Moreover, DS-CDK5-KD mice adopted a particular posture (curling up) that coincided with the time at which they were most likely to sleep during the test period (Fig. 2F and G). In addition, we examined the effect of DS-specific CDK5 KD on 48 h activity/rest behavior using the running wheel under LD and DD conditions. Control littermates exhibited a normal circadian activity of locomotion, whereas DS-CDK5-KD mice exhibited disordered circadian locomotor activity under the LD and DD condition (Fig. 3A and B). The activity/rest disturbance indicates that striatal CDK5 is largely associated with w circadian change.

CDK5 is an important Ser/Thr protein kinase that participates in actin-binding, synaptic morphology maintenance, and postsynaptic organization [37]. CDK5 also regulates the trafficking of synaptic vesicles and neurotransmitter release and contributes to homeostatic scaling [38, 39]. Consistently, in DS-CDK5-KD mice, we found changes in dendritic branching and spine formation, as well as altered CDK5-dependent phosphorylation of Tau at Thr181 in the DS region. Proper spine density and morphology and a balance between synaptic excitation and inhibition (E/I balance) are widely regarded to be essential for sleep–wake rhythms [40]. Furthermore, most E/I synapse in the brain are on dendritic spines, which are small protrusions on dendritic shafts that are important for synaptic plasticity [41, 42]. The striatum contains abundant GABAergic MSNs expressing the D_1_ receptor, which plays a key role in sleep–wake behavior [43, 44]. Here, we provided evidence that several GAD-positive neurons colocalized with CDK5 signals expressed in MSNs (Fig. 6B). In addition, whole-cell recording in DS-CDK5-KD mice revealed a specific decrease in GABA-mediated sIPSCs, whereas no change was observed in mEPSCs. Given that the frequency of sIPSCs reflects the release of presynaptic GABA, CDK5 likely modulates GABA receptor-mediated neurotransmission. The GABAergic system, particularly in MSNs, is the major inhibitory neurotransmitter system that underlies the E/I balance in the central nervous system. GABAergic neurotransmission dysfunction has been implicated in the pathogenesis of numerous behavioral conditions [45–48]. Therefore, CDK5 may perturb cytoskeletal assembly and spine density, affecting the GABAergic synaptic E/I balance in the striatum, reducing the inhibitory output of MSNs (Fig. 6C–F), and subsequently resulting in altered activity/rest behavior associated with circadian rhythms.

Brain functions are mediated by multiple neuronal activities involving highly elaborate and complex synaptic connections. Neurons need to transport organelles, proteins, and lipids from the soma to the axon and dendrites and back again to maintain a normal functional state [49, 50]. Microtubules act as conduits for both anterograde and retrograde transport of molecules [51, 52]. Here, we found that total Tau expression and Tau phosphorylation at Thr181 were significantly decreased in DS-CDK5-KD mice (Fig. 5C, D). Tau is highly enriched in neurons and was originally identified by its ability to bind to and stabilize microtubules. The equilibrium between Tau phosphorylation and dephosphorylation modulates the stability of the cytoskeleton and synaptic morphology in the normal brain. Consistent with this, we found that CDK5 KD perturbed the anterograde trafficking of AAV from the DS to the M2, thalamic nuclei, and BLA. Night-time sleep disturbances are common in PD, affecting up to 90% of PD patients [8]. The M2, thalamic nuclei, and BLA are closely related to sleep–wake rhythms. The M2 is important for processing and integrating sensorimotor cues and is involved in motor planning [53]. The thalamus is a critical node that integrates input and output in the central nervous system, and striatal-thalamic connections are the foundation of several higher brain functions [54, 55]. The BLA is the most important brain region for the circadian clock [56]. Thus, we propose that CDK5 dysfunction affected microtubule equilibrium and transport between the DS and other brain structures associated with sleep–wake rhythms.

In the current study, we did not examine whether CDK5 dysfunction is involved in night-time sleep disturbances associated with neurodegenerative disorders, such as PD or AD, and this remains to be addressed in future studies. However, in an animal model of PD, in which the neurotoxin MPTP is used to selectively induce the neurodegeneration of DA neurons in the SNpc, higher levels of CDK5 activity are detected [57]. Furthermore, in MSNs (the major neuronal type in the striatum), in which DA D_1_ and D_2_ receptors are generally considered to exert opposite effects at the cellular level [58, 59], stimulation with DA results in the opposite regulation of cAMP and PKA via stimulatory G protein (Gs/Golf)-mediated signaling in D_1_-MSNs compared with inhibitory G protein (Gi/Go)-mediated signaling in D_2_-MSNs [60–62]. Importantly, PKA activation increases calcium levels, leading to CDK5 activation, and increased CDK5-mediated phosphorylation of DARPP-32 at Thr75 is accompanied by decreased DA-D_1_-PKA-induced phosphorylation of DARPP-32 at Thr34 [16, 17]. We therefore conclude that the greater reduction in total striatal CDK5 may have a profound influence on the functions of D_1_-MSNs. Consistent, the selective involvement of CKD5 in D_1_-MSNs has been documented in the other studies [17, 63]. The striatum contains abundant GABAergic MSNs expressing the D_1_ receptors, which play a key role in sleep–wake behavior [43, 44]. Therefore, the current findings provide a foundation for extending our understanding of the role of CDK5 in the pathogenesis of sleep–wake rhythm disorders.

**Fig. 8:**
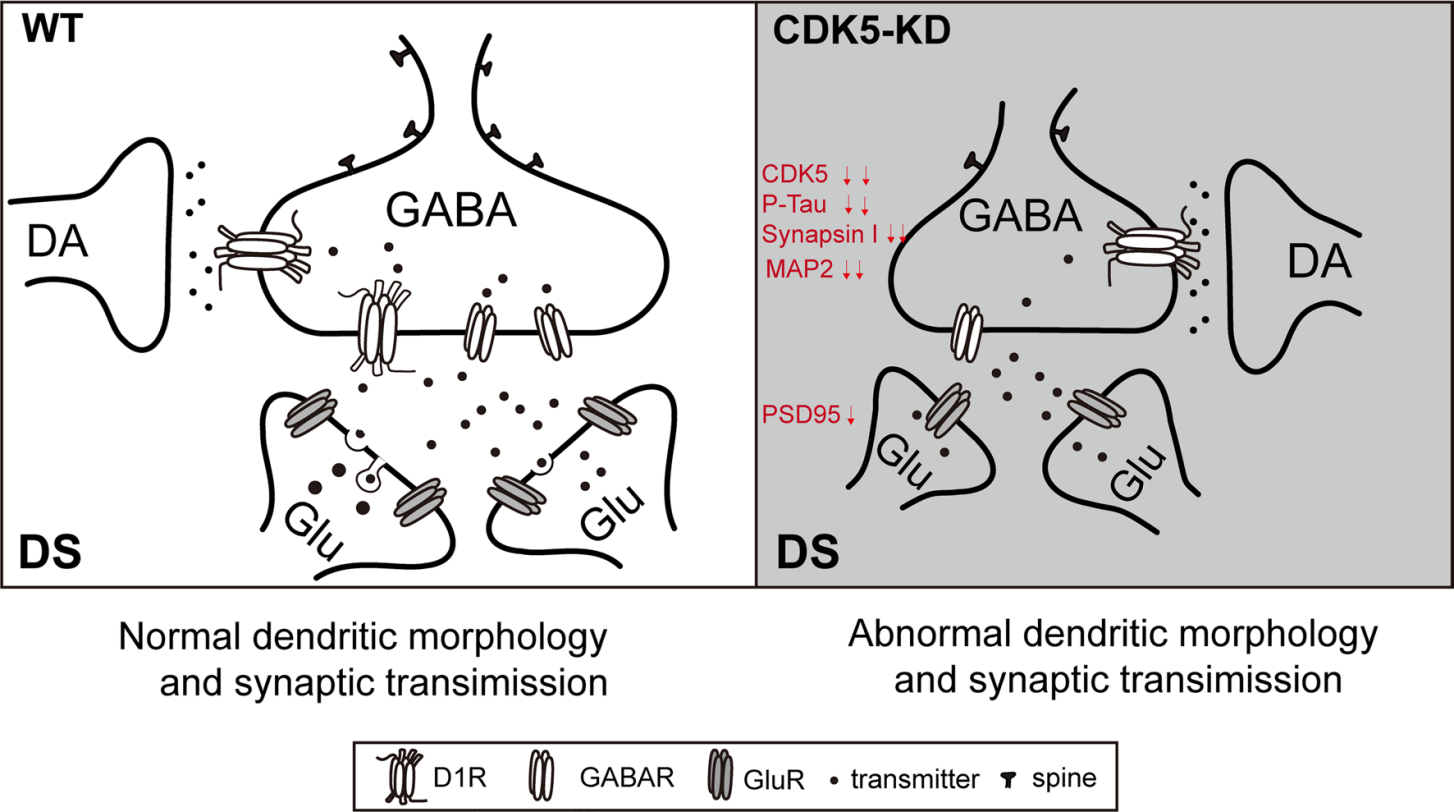
Proposed consequences of CDK5 loss on DS neuronal transmission. Compared with WT mice, shown on the left side, mice with CDK5 knockdown exhibit reduced dendrite length and synapse density associated with downregulation of MAP2, PSD-95, synapsin 1, and p-Tau Thr181 in the DS. This results in impaired neuronal signaling and reduced long-range projections from the DS to M2, thalamus, and BLA

Together, our findings demonstrate a pivotal role of CDK5 in inter-regional connectivity, neurotransmission, and motor control in the striatum. Targeted knockout of the *Cdk5* gene in the DS produced major changes in dendrite structure at most E/I synapse of MSNs in the striatum. These changes impacted communication between the DS and other brain regions, resulting in the dysregulated initiation of sleep–wake disturbance behaviors (summarized in Fig. 8). Collectively, our findings implicate that CDK5 in the DS plays a key role in the maintenance of striatal neural circuits associated with motor and circadian behavior in mice.

## Supplementary Information

**Additional file 1.** Additional methods.

**Additional file 2: Fig. S1. **(A) Cell morphology in the DS area was detected by HE staining. The striatal neuron in DS-CDK5-KD mice were morphologically normal and showed no significant loss. Scale bars = 20 μm. (B) Left panel: detrimental effects in the DS area of CDK5-KD were detected by Nissl staining. Right panel: quantitative profiles of Nissl bodies in the DS area of WT, DS-CDK5-NC, and DS-CDK5-KD mice. Nissl staining revealed no detrimental effects induced by the delivery of LV/Cas9-CDK5-sgRNA into the DS (Nissl body, WT: 7.467 ± 0.55, DS-CDK5-KD: 7.733 ± 0.51, DS-CDK5-KD: 6.733 ± 0.45). Arrows: Nissl body. Scale bars = 0 μm. (C) Left panel: cell apoptosis in the DS area was detected by TUNEL staining. Right panel: quantitative profiles of TUNEL-positive cells in the DS area of WT, DS-CDK5-NC and DS-CDK5-KD mice. Quantitative analysis for the TUNEL-positive cells showed there were no significant difference among in WT, DS-CDK5-NC, and DS-CDK5-KD mice (TUNEL-positive cell, WT: 2.47± 0.27, DS-CDK5-KD: 3.07 ± 0.27, DS-CDK5-KD: 3.33 ± 0.32). Arrows: TUNEL-positive cells. Scale bars = 20 μm. The TUNEL-positive cells showed the typical morphological features of apoptosis such as chromatin condensation, cytoplasmic budding and apoptotic bodies. Data are represented as the mean ± SEM, *n* = 15. One-way ANOVA, Tukey’s multiple comparisons test.

## Data Availability

All data generated or analyzed during this study are included in this published article and its additional information files.

## Abbreviations

AAV: Adeno-associated virus
ACSF: Artificial cerebrospinal fluid
AD: Alzheimer’s disease
BLA: Basolateral amygdala
CDK5: Cyclin-dependent kinase 5
CNQX: 6-Cyano-7-nitroquinoxaline-2, 3-dione
CRISPR: Clustered Regularly Interspaced Short Palindromic Repeats
DA: Dopamine
DARPP-32: Dopamine- and cAMP-regulated phosphoprotein of 32 kDa
DS: Dorsal striatum
GABA: γ-Aminobutyric acid
GAD: Glutamate decarboxylase
KD: Knockdown
l-AP5: L-(+)-2-amino-5-phosphonopentanoic acid
LV: Lentivirus
M2: Secondary motor cortex
MAP2: Microtubule associated protein 2
mEPSC: Miniature excitatory postsynaptic current
MPTP: 1-Methyl-4-phenyl-1,2,3,6-tetrahydropyridine
MSN: Medium spiny neuron
NC: Negative control
PD: Parkinson’s disease
PSD-95: Postsynaptic density protein 95
SgRNA: Small guide RNA
sIPSC: Spontaneous inhibitory presynaptic current
SN: Substantia nigra
TTX: Tetrodotoxin
WT: Wild type
