# Supplementary material for: Downregulation of CDK5 signaling in the dorsal striatum alters striatal microcircuits implicating the association of pathologies with circadian behavior in mice
Source: Mol Brain. 2022 Jun 14;15:53. doi: 10.1186/s13041-022-00939-2 (PMC9195255; doi:10.1186/s13041-022-00939-2)
Supplement: Supplementary file 1 — Additional file 1. Additional methods. [file 13041_2022_939_MOESM1_ESM.docx]

## Additional methods

## Hematoxylin and eosin (HE) staining

Mouse brains were quickly removed, rinsed, fixed in 4% paraformaldehyde in 0.01 mol/L PBS (pH 7.4) at 4°C for 48 h, dehydrated, embedded in paraffin, cut into 5-µm sections with a microtome (Leica, Wetzlar, Germany), and mounted onto gelatinized slides. All slides were washed twice with distilled water for 2 min before being immersed in hematoxylin solution (Beyotime Biotech, Jiangsu, China, #C0105S) for 5–10 min. Slides were then washed with distilled water and stained with eosin solution (Beyotime Biotech, Jiangsu, China, #C0105S) for 1 min. Slides were rinsed for 3 min in distilled water, dehydrated in ascending alcohol solutions (50%, 70%, 80%, and 95% × 2, followed by 100% × 2) and sealed with nail varnish.

## Nissl staining

Nissl staining was performed using a Nissl staining kit (Beyotime Biotech, Jiangsu, China, #C0117) following the manufacturer’s instructions. The coronal sections were stained with the Nissl staining solution for 5 min and washed with PBS for 10 min. Finally, the sections were serially dehydrated sequentially in 50%, 75%, 95%, and 100% ethanol and sealed with neutral gum. Three fields were randomly chosen at 400× magnification for five brain sections, and the number of Nissl bodies was counted in a 1-mm length of the DS under light microscopy (Nikon, Tokyo, Japan).

## TUNEL staining

Cell death was detected using a terminal deoxynucleotidyl transferase-mediated dUTP nick end labeling (TUNEL) kit (Beyotime Biotech, Jiangsu, China, #C1098) following the manufacturer’s instructions. Brain sections were prepared and incubated with the terminal deoxynucleotidyl transferase (TdT) enzyme and reaction mixture at 37°C for 1 h in a wet box. The converter-POD was added, and sections were incubated in a humid chamber at 37°C for 30 min. After coloration using diaminobenzidine (DAB) chromogenic agents at room temperature for 10 min, the nuclei were stained using hematoxylin. TUNEL-positive cells exhibited fluorescence by incorporating fluorescein-12-dUTP at the 3'-OH ends of fractured DNA after catalysis induced by TdT. Pictures were taken using a light microscope (Olympus). Three fields were randomly chosen at 400× magnification for five brain sections, and the number of TUNEL-positive cells was counted in a 1-mm length of the DS under light microscopy (Nikon, Tokyo, Japan).
